# Influence of Illumination on Porous Silicon Formed by Photo-Assisted Etching of p-Type Si with a Different Doping Level

**DOI:** 10.3390/mi11020199

**Published:** 2020-02-14

**Authors:** Olga Volovlikova, Sergey Gavrilov, Petr Lazarenko

**Affiliations:** Institute of Advanced Materials and Technologies, National Research University of Electronic Technology (MIET), Moscow 124498, Russia

**Keywords:** photo-assisted etching, porous silicon, illumination, doping level, total current, reflectance

## Abstract

The influence of illumination intensity and p-type silicon doping level on the dissolution rate of Si and total current by photo-assisted etching was studied. The impact of etching duration, illumination intensity, and wafer doping level on the etching process was investigated using scanning electron microscopy (SEM), atomic force microscopy (AFM), and Ultraviolet-Visible Spectroscopy (UV–Vis–NIR). The silicon dissolution rate was found to be directly proportional to the illumination intensity and inversely proportional to the wafer resistivity. High light intensity during etching treatment led to increased total current on the Si surface. It was shown that porous silicon of different thicknesses, pore diameters, and porosities can be effectively fabricated by photo-assisted etching on a Si surface without external bias or metals.

## 1. Introduction

Porous silicon (por-Si) has been receiving a great deal of attention due to its interesting physical and optical [1] properties and promising potential technological applications in the fields of sensing [2,3], Li-ion batteries [4], and solar cells [5]. The morphology of the silicon wafer may be modified by electrochemical wet etching [6,7,8], galvanic etching [9,10,11], or metal-assisted chemical etching (MACE) [12,13] to produce pores, nanowires, and microstructures. These methods have already been studied in detail. A cheap, simple way to make porous silicon layers without surface contamination is important for silicon technology. It is possible to produce porous silicon layers in solutions containing HF and H_2_O_2_ under illumination without external bias or a metal film on the Si surface. This technique is the photo-assisted etching of Si (PhACE). The silicon wafer is not connected to the external power supply, and the electric field is due to band bending. PhACE can produce porous silicon for solar cell applications; however, this method is poorly understood. The physical properties of porous silicon are determined by two large groups of factors that affect carrier density on the surface. The first group of factors includes doping type and charge carrier concentration [10,14]. The second group includes illumination during etching [15,16]. The influence of lamp illumination on the properties of n-type porous silicon samples during the etching process has been deeply investigated. For example, Koker and Kolasinski [17] investigated laser-assisted porous silicon formation on n-Si. In addition, several authors have investigated the photodissolution of n-type silicon in HF-containing electrolyte [7,18]. On n-type silicon, illumination is used to supply holes at the silicon surface and for pore growth. It depends on illumination intensity and wavelength. On p-type silicon, illumination may increase the conductivity of the substrate and of the porous layer [19]. Most of the present observations can be rationalized in terms of a photoinduced etching mechanism [20]. p-Type Si (p-Si) can also be modified by different illumination intensities [16]. The concentration of charge carriers and illumination intensity regulates the current density in the etching process. The current density on the silicon surface not only determines the rate of por-Si formation but also has a significant impact on its structure and physical characteristics. PhACE is characterized by low silicon etching rates, economic efficiency, and the ability to etch many wafers simultaneously. Only the uniform supply of reagents and light to the surface of silicon is required. There is no data on the etching rate and charge on the Si surface during PhACE of p-Si under visible light illumination in the literature. The originality of this paper is the formation of porous silicon and an investigation of the etching rate and total current on the Si surface during etching under front-side illumination and the dark. The paper provides information about the features of the porous silicon formation process in a potentiostatic regime.

## 2. Materials and Methods

Wafers of p-Si (100) with ρ = 0.01, 1, and 12 Ω·cm were used as the substrate. The silicon wafers were treated with piranha etch (a mixture of H_2_SO_4_ (98 wt.%) and H_2_O_2_ (30 wt.%) (1:2 by volume)) for 600 s at 130 °C. Then, the wafers were rinsed with deionized water and dried in an isopropyl alcohol vapor flow. The organic solvent vapor reduces the surface tension of the solution, and, due to this, water and small particles are removed from the surface of the substrate.

After that, the Si wafers were rinsed in an HF-H_2_O solution (1:5 by volume) to remove native SiO_2_. The wafers were then cut into pieces and used as samples. The etching was performed in a solution that contained HF (40 wt.%):H_2_O_2_ (30 wt.%):H_2_O (2:1:10 by volume) at 25 °С for 20, 40, 60, and 80 min. The treatment was performed in the dark in an opaque Teflon beaker. The treatment was also performed with visible light using front-side illumination from a halogen lamp (JCDR 50 W) equal to 460 and 8000 lx. The distance between the lamp and the sample was 40 cm. The solution temperature was constant during illumination. The entire nonworking surface was protected by a chemically resistant varnish during etching. The entire working area was illuminated. Then, the samples were rinsed in an ethanol–water solution and dried in air. The sample surface morphology was investigated by scanning electron microscopy (SEM) using a JSM-6010PLUS/LA (JEOL company, Peabody, MA, USA) and Helios NanoLab 650 (Thermo Fisher Scientific, Waltham, MA, USA). AFM measurements were carried out in non-contact mode using a silicon cantilever at a resonance frequency of about 200 kHz under ambient conditions (microscope SOLVER-PRO, NT-MDT Ltd., Moscow, Russia) for roughness and pore walls analysis. AFM measurements were analyzed in Image Analysis. Gravimetric analysis was used for the porosity of the porous silicon calculation. The samples (five samples) were weighed before (*m_1_*) and after (*m_2_*) etching using analytical balance XP 205 (Mettler Toledo, Greifensee, Switzerland). The average values of the masses and the standard deviation were calculated:

$$\bar{m_{n}}=\frac{m_{n1}+m_{n2}+\ldots +m_{n5}}{5},\;\Delta m=\sqrt{\frac{\sum_{i=1}^{5}{\left(m_{ni}-\bar{m_{n}}\right)}^{2}}{4}}.$$

The sample area was measured (*S* = *a* × *b*, *a*—length of the sample, *b*—width of the sample). It ranged from 0.5 to 1.5 cm^2^. After etching, the porous silicon was dissolved in a 1 wt.% NaOH aqueous solution at room temperature until gas evolution stopped. After drying at room temperature, the samples were weighed (*m_3_*). Porosity was calculated by the following equation:

(1) $$P=\;\frac{m_{1}-m_{2}}{m_{1}-m_{3}}\times 100\%,$$

where *m_1_* and *m_2_* are the sample’s masses before and after etching and m_3_ is the sample mass after por-Si dissolution. Samples were etched in the same conditions because of the multi-sectional Teflon cell. 

The short-circuit current in the galvanic cells was measured with a digital multimeter (UNI-T UT61C, Hong Kong, China). A Si sample connected with a Pt-electrode and set to a HF and Н_2_О_2_-containing solution formed a galvanic cell. The value of the charge *Q’_Excess Carrier_* was determined by numerical integration of current versus time, *Q =*$\;\int_{0}^{t}Jdt$. The optical properties of the porous silicon formed by PhACE were measured using a UV–Vis–NIR spectrophotometer (Cary 5000, with an integrating sphere) in the 200–1300 nm wavelength range. Surface reflectance measurements were used to confirm that the porous silicon produced was useable for solar cells. 

Gravimetric analysis was also used to define the number of carriers consumed over seconds during photo-assisted chemical etching. The investigations of excess current through silicon during PhACE with different illumination intensities were carried out using the short-circuit current. Short- circuit measurements were used to define current through the wafer during photo-assisted chemical etching. A mechanism for the formation of porous silicon without an external bias using the short-circuit measurement, *J(t)*, was established.

## 3. Results

It was found that porous silicon formation on p-Si is possible using front-side illumination without an external current source (electrochemistry) or metal on the surface of Si (MACE). The Si-HF-H_2_O equilibrium diagram in [21] indicates band bending of p-Si in an HF solution, which leads to a hole-depleted layer formation on the Si surface. Therefore, a potential greater than the anodic silicon decomposition potential is required. Therefore, silicon anodic dissolution reactions are provided either by applying an external voltage, the deposition of a metal on the Si surface, or illumination. Photon absorption excites electrons to the conduction band, resulting in the formation of valence band holes. Silicon does not dissolve without an oxidizer (H_2_O_2_), because the role of H_2_O_2_ under photon excitation is to consume the excited conduction band electrons. The electrochemical potential of H_2_O_2_ is much more positive than the valence band of Si, and hole injection from H_2_O_2_ into the valence band is energetically possible [12,22]. This facilitates the transport of holes to the Si surface, which can then induce etching. 

The mechanism of the photo-assisted etching of p- and n-Si with *ρ* = 2 Ω·cm in HF was described in [23], with *ρ* = 0.01 and 10 Ω·cm [24]. Peroxide reduction is cathodic and silicon dissolution is an anodic process [25]:

(2) $$H_{2}O_{2}+2H^{+}+2e^{-}\to 2H_{2}O,$$

(3) $$Si+6HF\to SiF_{6}^{2-}+6H^{+}+4e^{-}.$$

Por-Si formation on the Si surface is possible due to the heterogeneous distribution of the electrochemical potential. There are some places on the surface of Si near which dissolution occurs at a higher rate. Doping level and resistivity influence the rate of pore formation. The lower the resistivity, the faster the etching rate. With the same modes of Si etching, silicon resistivity affects the amount of dissolved silicon. Additional illumination allows hole generation due to photon absorption. Porous silicon is the result of photo-assisted p-Si etching.

**Figure 1:**
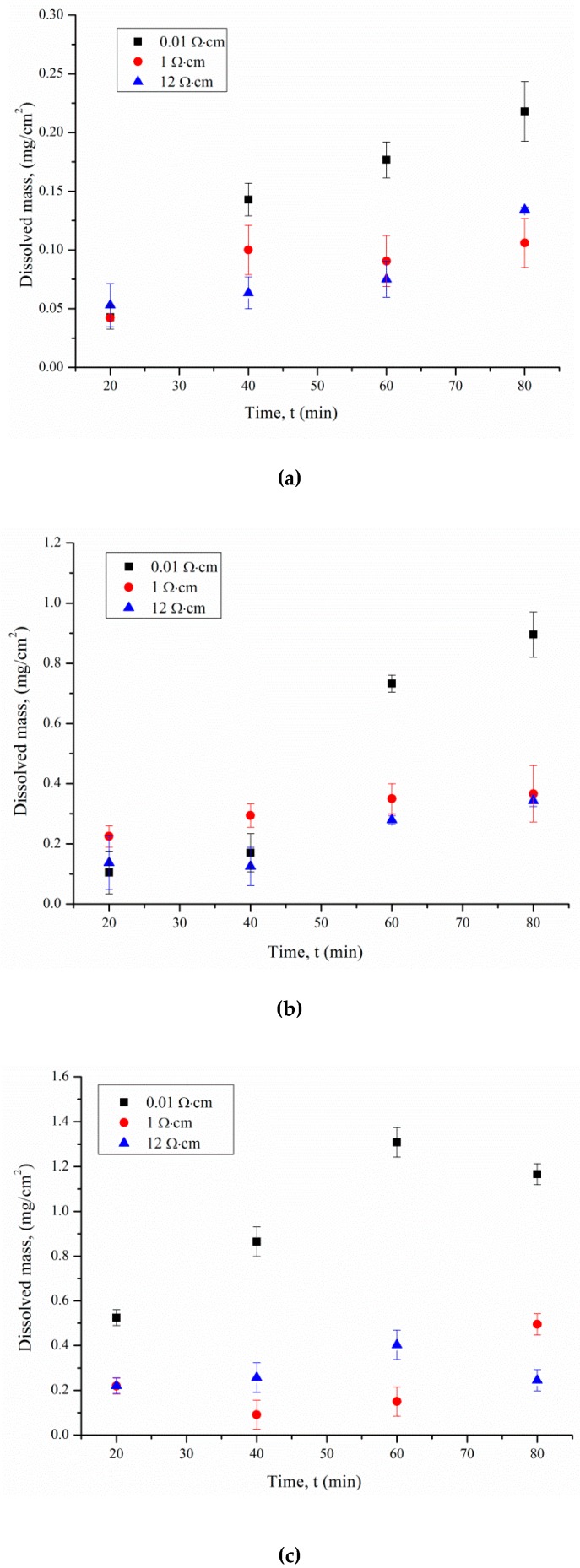
The effect of treatment duration on dissolved Si mass per surface area (**a**) in the dark, and under (**b**) 460 and (**c**) 8000 lx illumination.

Figure 1 shows the time dependence of dissolved p-Si with 0.01, 1, and 12 Ω·cm for different illumination intensities (Δ*m* = *m_1_* − *m_2_*). The error was calculated as the standard deviation.

The effect of the resistivity, *ρ*, of doped silicon on the rate of its dissolution was noted. This effect is due to two factors. The inhomogeneous electrochemical potential distribution on the surface of the wafer is the first. The potential dispersion increases with impurity concentration in Si, so the pore density increases. Therefore, for p-Si with a lower resistivity, the dissolved mass will be higher than for p-Si with a high resistivity.

The silicon dissolution rate is directly proportional to the illumination intensity. The smaller value for the dissolved silicon mass at 0 lx is due to the low charge carrier generation rate at the Si surface in contact with the electrolyte. The maximum etching rate of Si was observed for p-Si with *ρ* = 0.01 Ω·cm under 8000 lx illumination. The rate of Si dissolution for *ρ* = 0.01 Ω·cm is 3 and 11.8 μg/min, that for *ρ* = 1 Ω·cm is 1.2 and 3.9 μg/min, and that for *ρ* = 12 Ω·cm is 3 and 0.9 μg/min in the dark and under 8000 lx illumination, respectively. We approximated the dependence of the dissolved silicon mass change Δ*m* = *m_1_* − *m_2_* on the etching time by a linear function. The angular coefficient of the linear approximation allowed the silicon etching rate for the solution to be calculated.

Front-side illumination is necessary for porous silicon formation. The etching rate is determined by hole (h+) accumulation in the contact area of the HF electrolyte and Si atoms. The hole generation rate (*g_x_*) in a semiconductor is given by the following equation [26]:

(4) $$g_{x}=I_{0}\cdot \alpha \cdot e^{-\alpha \cdot x},$$

where *I*_0_ is the intensity of light during etching, *x* is the depth of the light penetration, and *α* is the absorption coefficient.

**Figure 2:**
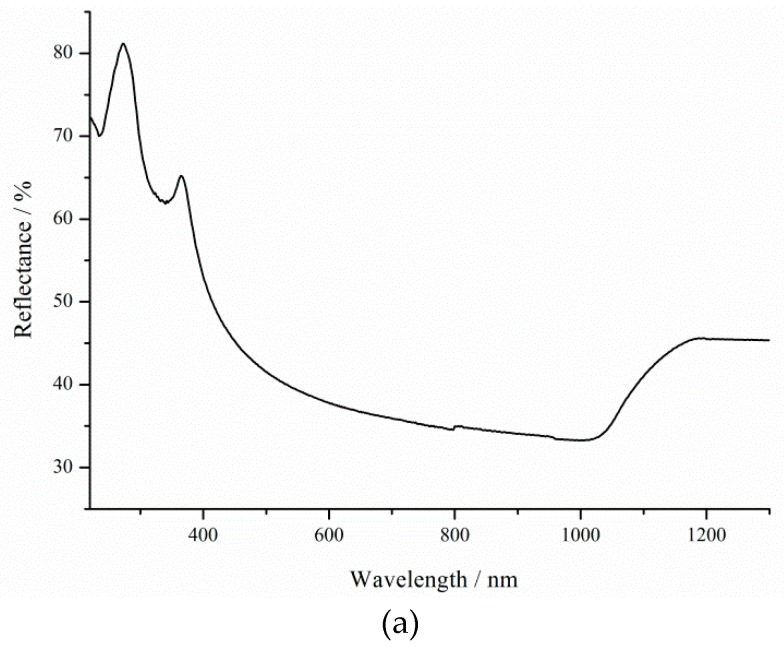

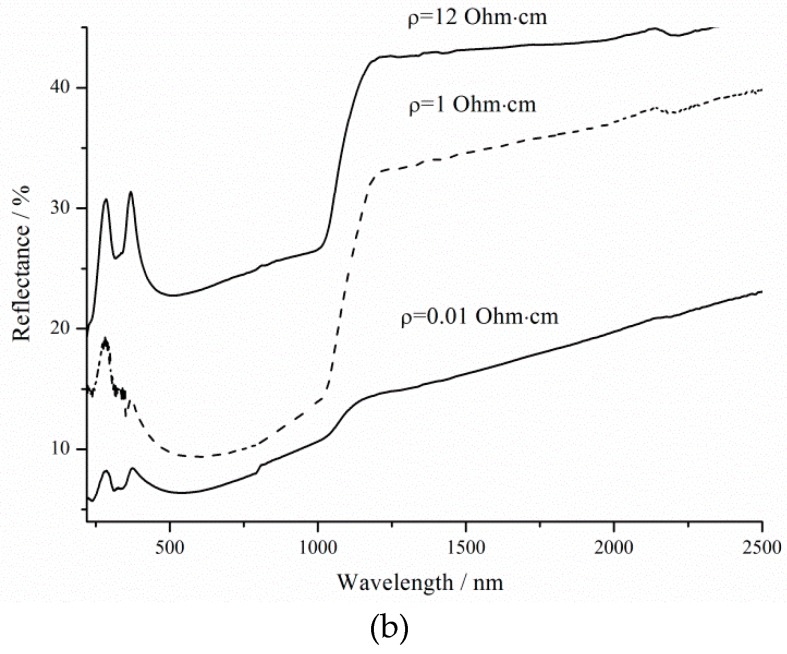
(**a**) Reflectance curve of the sample with *ρ* = 0.01 before treatment, and (**b**) reflectance curves of the samples with *ρ* = 0.01, 1, and 12 Ω·cm after 10 min of treatment.

According to Equation (4), the first factor that affects the hole generation rate is the light intensity during etching. The higher the illumination intensity, the higher the dissolution rate (Figure 1). Light absorptance (LA) is the second factor that affects the hole generation rate. Silicon wafers with *ρ* = 0.01, 1, and 12 Ω·cm before photo-assisted etching have a low absorption coefficient. The effect of short-term silicon etching in the solution containing HF and H_2_O_2_ is por-Si, which exhibits high LA properties [16,26] (Figure 2). LA depends on porous silicon thickness and pore diameters. Etching rate and resistivity influence these.

Figure 2 shows the reflectance spectra of silicon before etching and porous silicon formed for 600 s under 8000 lx illumination.

As can be seen from the reflectance spectra, even after 600 s of porous silicon formation, the optical reflectance at the air–silicon interface is significantly reduced as the porous layer thickness increases. The resistivity influences the porous silicon thickness (Figure 1). Thus, the sample formed on p-Si with *ρ* = 0.01 Ω·cm under 8000 lx illumination has a minimum reflectance, because the pore density has maximum values in this case. Porous silicon thickness depends on treatment duration and illumination.

The dissolution rate of Si is a function of the current flowing through the circuit, which characterizes the total number of charge carriers that are injected into silicon and participate in its dissolution over seconds. The total current (TC), *J*, on the Si surface during etching in the dark and under illumination can be calculated as follows [27]:

(5) $$J=\frac{\frac{\left(m_{1}-m_{2}\right)}{S}\cdot n\cdot e}{t\cdot m_{Si}}=\frac{V\cdot n\cdot e}{m_{Si}},$$

where *m_1_* is the mass before etching, *m_2_* is the mass after etching during *t*, (*m_1_*− *m_2_*)/*S* is result of gravimetric analysis, *m_Si_* is mass of the Si atom, *n* is the number of holes required for dissolution of each silicon atom, which equals 2 [28], *t* is the etching duration, *e* is the elementary electric charge, and *S* is the sample area.

**Figure 3:**
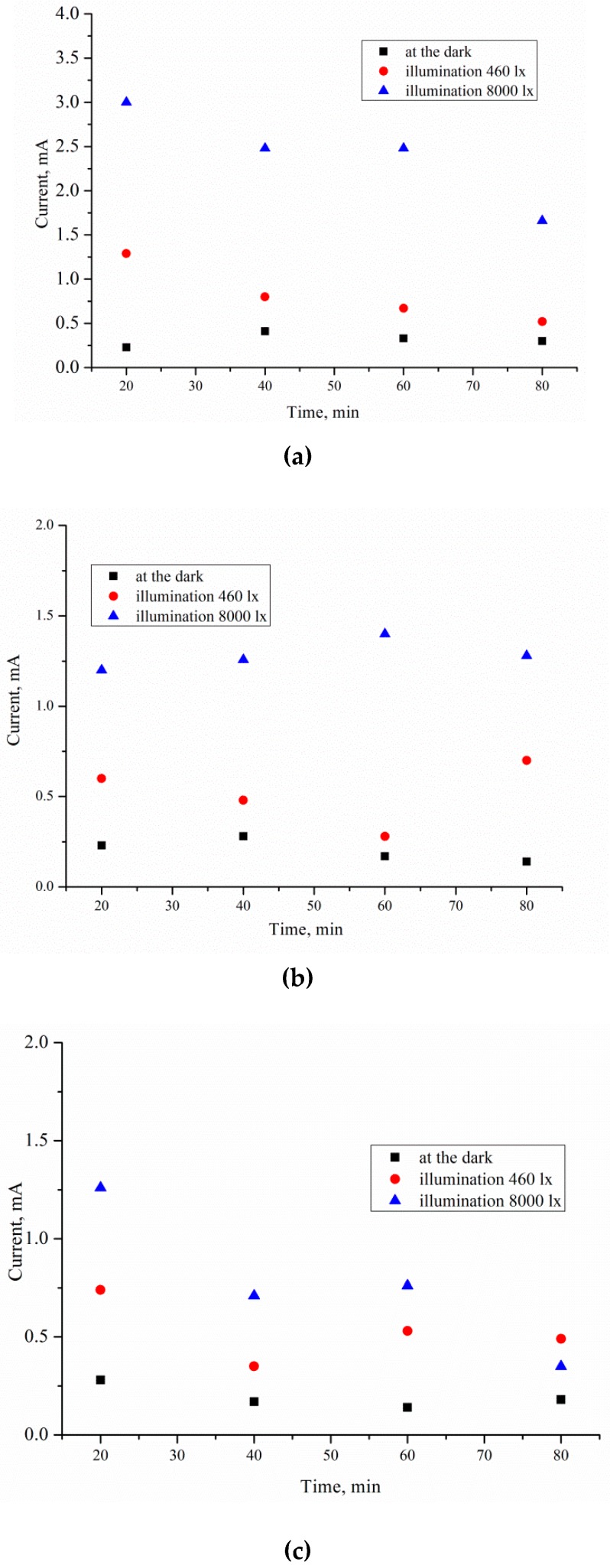
Current density, *J*, on the Si surface equaling 1 cm^2^ after etching silicon with: (**a**) *ρ* = 0.01, (**b**) 1, (**с**) and 12 Ω·cm.

Figure 3 shows that the TC, *J*, calculated using Equation (5), occurs on the p-Si surface during PhACE under different illumination intensities. The current decreases with increasing treatment time as the rate of porous silicon formation slows down. There are two possible reasons. First, the porous silicon layer absorbs light. Light does not reach the bottom of the pores, which leads to dissolution of the pore walls but not growth of the porous layer thickness. The second reason is that with an increase in thickness of the porous layer, reagents take longer to reach the chemical reaction zone at the bottom of the pores, so the concentration of HF and H_2_O_2_ decreases, as does the rate of silicon dissolution. This fact explains the form of the *J(t)* curve.

TC increases with the front-side surface illumination intensity. Light is suitable for creating majority charge carriers (holes) in silicon, which are necessary for etching. Thus, the values of the anode *J* depend on charge transfer mechanisms controlled by the illumination level and the resistivity of the initial Si wafer.

**Figure 4:**
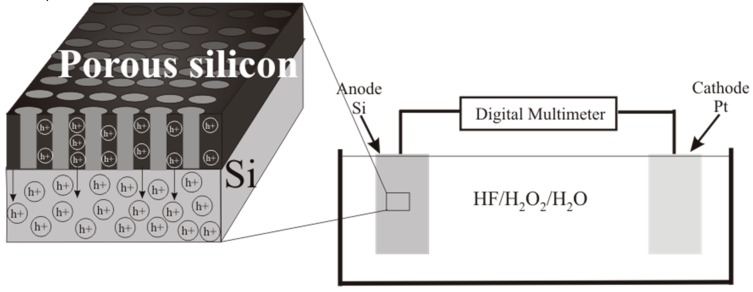
Schematic of the galvanic cell.

A p-type silicon wafer connected to platinum in a solution containing HF and H_2_O_2_ gives a galvanic cell [29,30]. Pt is in the electrolyte close to the sample surface. The current flows through the electrolyte between the Pt cathode and the silicon anode. This current can be measured using the short-circuit current. The cell produces a steady-state current proportional to the silicon area (Figure 4).

**Figure 5:**
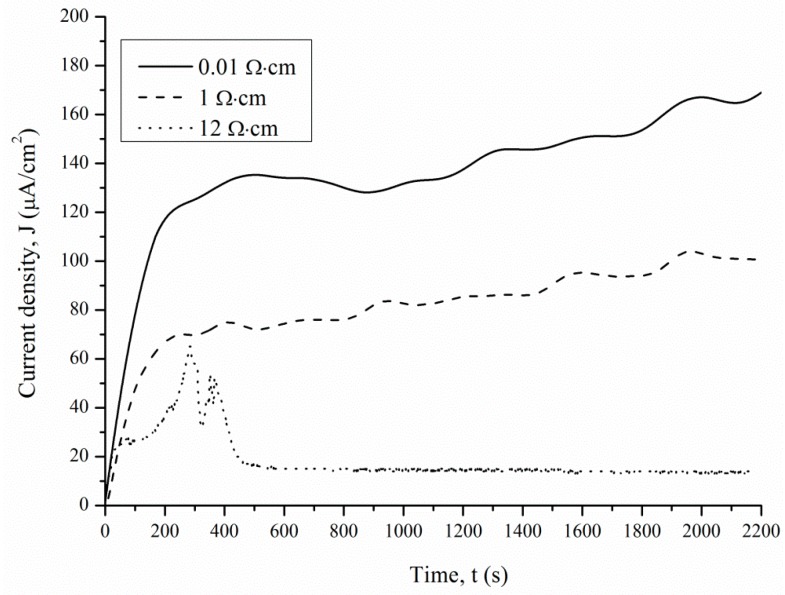
Current density measured in the dark.

**Figure 6:**
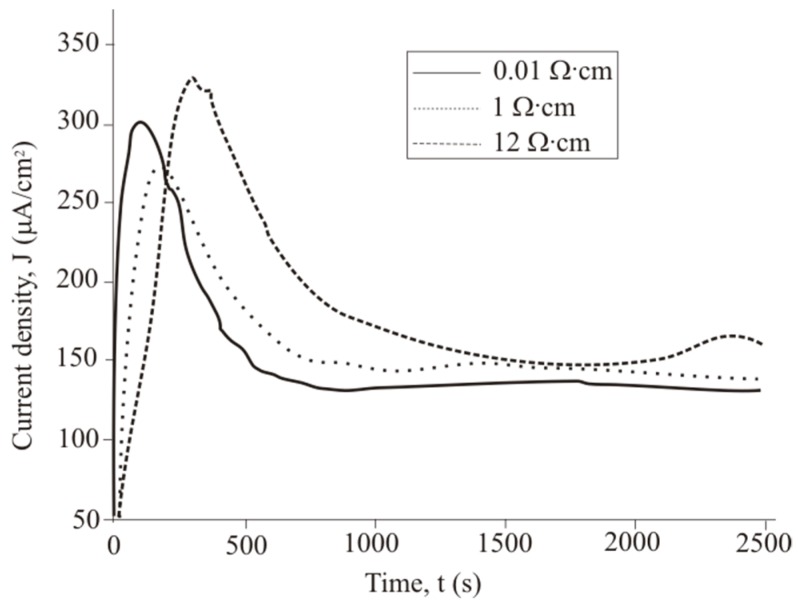
Current density measured under 460 lx illumination.

When the p-type silicon sample is connected to platinum in the HF/H_2_O_2_ solution, short-circuit current is observed in the dark and under illumination. Hydrogen peroxide is reduced during the etching. The holes injected into the valence band of silicon participate in the dissolution of Si and the formation of a porous layer. The transport of holes through porous silicon is difficult due to the high resistivity of the porous layers [31]. The excess holes that are not involved in the dissolution of Si diffuse into the semiconductor. The current of nonequilibrium charge carriers and the ion current can be measured by a galvanic cell (Figure 5 and Figure 6). Figure 5 and Figure 6 show the current measured after the Si and Pt are short-circuited in the dark and under illumination, respectively, for a 1 cm^2^ sample.

## 4. Discussion

The *J(t)* curve measured under 460 lx illumination has a typical shape for the potentiostatic regime. The short-circuit current occurring under 460 lx illumination first rises sharply and then decreases significantly over 180–360 s. After 360 s, the current density is reduced from the maximum value to a stable value.

The *J(t)* curves characterize the etching mechanism. Three characteristic regions can be identified:

1. Current increasing,
2. slowing growth rate and subsequent decrease in current,
3. constant current.

**Figure 7:**
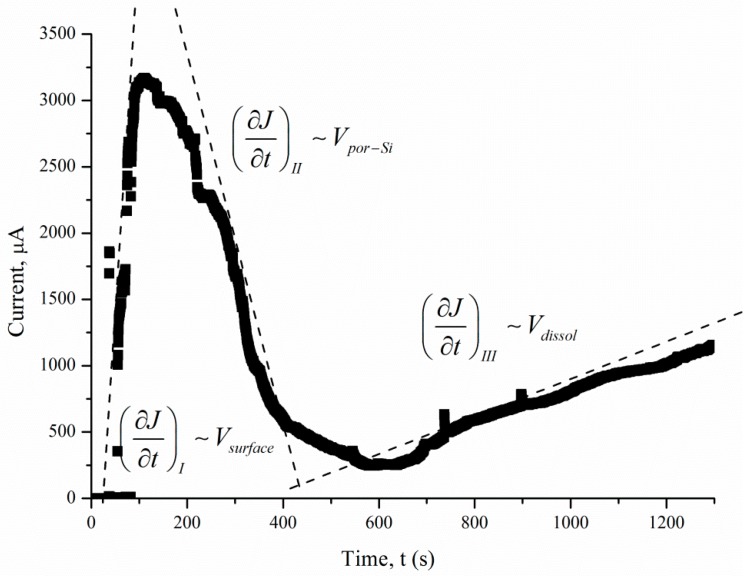
Current density measured under 8000 lx illumination.

*J(t)* reflects a change in the area (*S*) of the electrochemical reaction front. Changes in *J* with time (Figure 7) are related to the evolution of the Si morphology during pore nucleation. Pore formation takes place after the immersion of silicon into the solution containing HF and H_2_O_2_. The pore area depends on the duration of treatment. The increase in the specific surface area leads to growth of the current density—the first region. The current growth will continue until a porous layer of critical thickness is formed on the Si surface. In this case, access of the solution to the surface of monocrystalline silicon becomes limited. The transport of holes through porous silicon is difficult due to the high resistivity of the por-Si.

With an increase in the thickness of the porous layer, the concentration of excess holes that are not involved in the dissolution of Si becomes smaller, which is reflected in the value of the slope angle of region II, which characterizes the growth rate of the por-Si layer. A further effect of the solution on the surface leads to the dissolution of the porous layer (the beginning of region III), and the illumination of the sample will play an important role. Dissolution of por-Si will reduce the thickness of the porous layer to less than the critical value, which will increase the concentration of holes in Si and, consequently, the current in region III. The slope angle of the section will characterize the dissolution rate of the porous *V_dissol_* layer. That is why the inclination angle of region III *J(t)* measured under 460 lx (Figure 6) is less than that under 8000 lx illumination (Figure 7).

The slope of the I segment *(∂J/∂t*) characterizes the porous layer nucleation rate, which will be higher for *ρ* = 0.01 Ω·cm in the case of different values of *ρ*. This result is consistent with the experimental data in Figure 6. The higher the speed of the *V_surface_*, the greater the area of silicon dissolution, so a larger number of charge carriers will inject into silicon and reach the non-working side of the plate.

When comparing the results obtained from calculations using Equation (5) and measurements in a galvanic cell, a significant difference in currents was found. The value of *Q’_Excess Carrier_* was determined by numerical integration of the dependence of the current on time (Figure 6). The *Q’_Excess Carrier_* value was low compared to *Q_total_* as it characterizes the current of excess charge carriers that have diffused into the substrate. Table 1 presents the charge values *Q_total_, Q’_Excess Carrier_*, and Δ*Q* for samples with different treatment parameters.

The charge passing through the substrate is a term in Equation (6) and depends on the concentration of charge carriers injected into silicon *Q _total_*.

*Q’_ Excess Carrier_ = Q _total_ – Q.* (6)

*Q_total_*, calculated by Equation (5): *Q* = *J*·*t*, characterizes the value of all charge carriers involved in the etching process. The increase in charge carrier concentration on the surface of the hole caused by injection leads to the appearance of a diffusion electron flow directed along the x-axis perpendicular to the semiconductor surface, with the result that the carrier concentration increases not only on the surface but also in the depth of the semiconductor. In this case, injected carriers go deeper into the semiconductor at different distances, where they are recombined. The contribution of the morphology of the porous layer is significant in the value of *Q’_Excess Carrier_* as some of the charge carriers remain in the porous layer Δ*Q*.

Having obtained the values of Δ*Q*, it is possible to determine the effect of light intensity and resistivity to estimate the change in the thickness of the porous layer. The decrease in Δ*Q* with an increase in the resistivity during etching in the dark and 460 lx illumination is associated with a decrease in the thickness of porous silicon. This is confirmed by the results of gravimetric analysis. The high value of *Q’_Excess Carrier_* and low Δ*Q* samples etched at 8000 lx, in relation to the above, are associated with an intensive dissolution of the porous layer, as well as an increase in the diameter of the pores in the latter. The increase in the diameter of pores contributes to the improvement in the access of the reagents to the silicon surface, which increases the concentration of charge carriers injected into the semiconductor.

**Figure 8:**
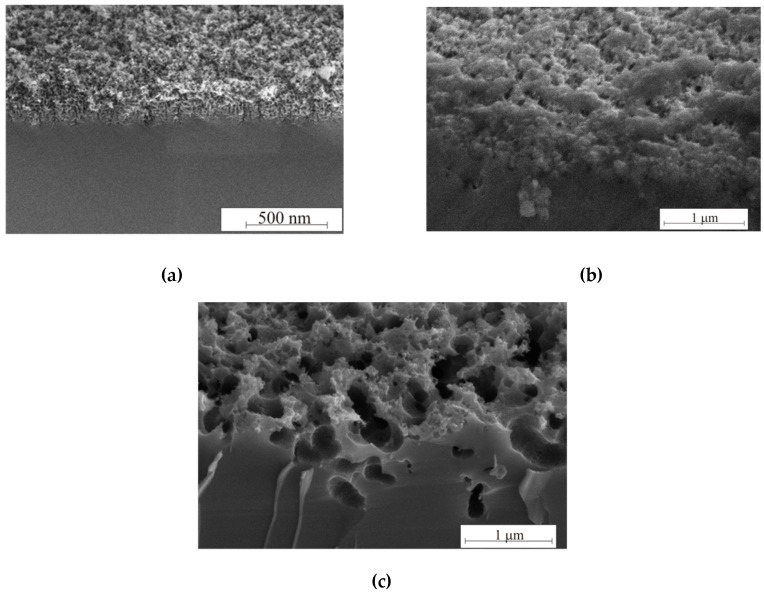
Cross-section of samples formed in the (**a**) dark, and (**b**) 460 and (**c**) 8000 lx.

As can be seen from the SEM images in Figure 8, the thickness of porous silicon formed in the dark reaches values not exceeding 300 nm, at 460 lx—not more than 1 micron, and at 8000 lx—the dissolution of the porous layer is clearly observed in both thickness and in the pore walls thickness. Illumination of the reaction zone is necessary for the thickness and porosity of porous silicon growth. However, long-term 8000 lx illumination contributes to the pore diameter growth, which leads to a reflectance increase.

**Figure 9:**
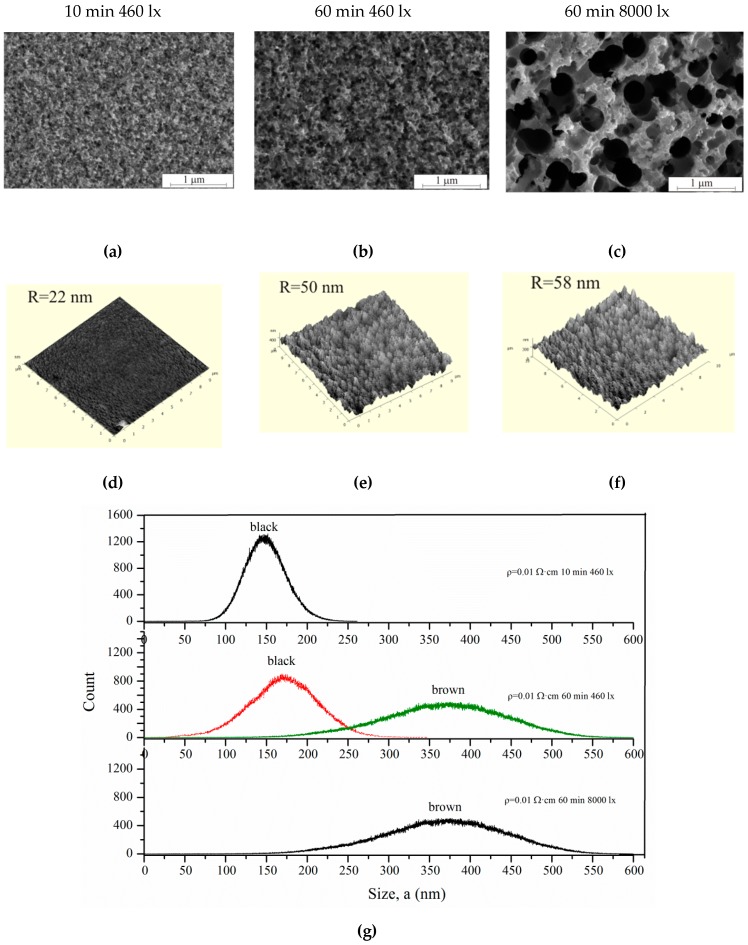
Morphology evolution of porous silicon by photo-assisted etching inspected after (**a**, **d**) 10 and (**b**, **c**, **e**, **f**) 60 min under (**a**, **b**, **d**, **e**) 460 and (**c**, **f**) 8000 lx illumination. (**a**–**c**) SEM images, (**d**–**f**) AFM images, and the (**g**) wall thickness of black and brown silicon.

Figure 9 shows SEM (a–c) and AFM images (d–f) of the samples with *ρ* = 0.01 Ω·cm after different etching regimes. The pore diameter increases after etching under high 8000 lx illumination.

In this case, the intense generation of holes occurs in the pore walls, which contributes to wall dissolution. The walls dissolve, and the pores gradually unite with each other. Such samples differ from black porous silicon (3600 s, 460 lx); they are brown. «Black» and «brown» porous silicon are characterized by the color of the samples. The roughness and pore wall size of the black and brown silicon were measured by AFM using the non-contact mode. AFM measurements were analyzed in Image Analysis, resulting in the roughnesses presented in Figure 9d–f. The thickness of the walls of black and brown silicon is shown in Figure 9g.

As can be seen from Figure 9 and Table 2, the pore sizes increase with treatment duration from 10–100 to 25–125 nm for 10 and 60 min, respectively. The illumination 8000 lx during the 60 min etching of silicon leads to the formation of a porous layer with 90–440 nm pore diameter. Thus, illumination intensity significantly influences the pore size. The illumination intensity and treatment duration influence the pore wall thickness. This is confirmed by AFM measurement results. Black porous silicon has a wall thickness of 150–175 nm; brown porous silicon has a 350–375 nm wall thickness, because of wall dissolution and porous under-layer formation.

**Figure 10:**
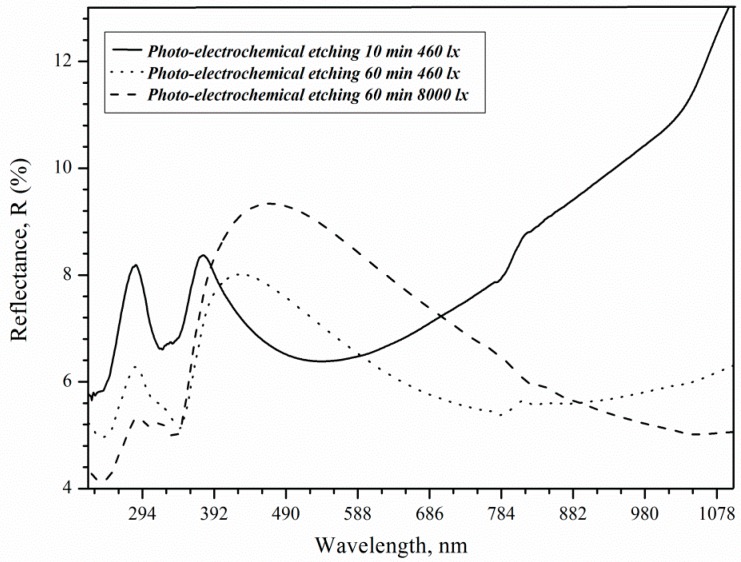
Reflectance curves of the samples with *ρ* = 0.01 Ω·cm after 10 and 60 min treatment under 460 and 8000 lx illumination.

The increase in roughness and porosity contributes to the deterioration of the optical properties of porous silicon for solar cell applications (Figure 10). In the visible (VIS) region, the refractive index decreases with porosity, in contrast to the increasing edge absorption coefficient with an increase in porosity [32]. The porous silicon layer further increases the efficiency and absorbs more light energy in c-Si solar cells [33].

Reflection peaks (8.4%) 280 and 370 nm are associated with direct interband transitions in c-Si in the case of 73 nm thick porous layers [34]. At these wavelengths, the reflectance decreases to 6.4 and 5.4% as the thickness increases due to rising treatment duration. The effect of changing the thickness is now critical for shorter wavelengths [35]. The reflection peak at 370 nm shifts along the x-axis to 425 and 480 nm for samples after 3600 s etching under 460 and 8000 lx illumination, respectively. The roughness of the surface can influence the shift in the position of the maximum. Hence, it can be concluded that the reflectance increase in the visible spectral range, as shown in Figure 8, is solely the result of the surface roughness induced by illumination, which is consistent with literature results [36]. As a result, an 8000 lx illumination of silicon during etching promotes an increase in the roughness and reflection of the porous silicon surface, which is then called brown.

Thus, a comprehensive study of the currents involved in the dissolution of silicon, short-circuit currents, and optical and morphological properties made it possible to identify the effect of illumination intensity and resistivity on the concentration of nonequilibrium charge carriers. The absence of an external source of current and a metal catalyst on the surface made it possible to establish a significant contribution to the illumination intensity and resistivity as separate parameters of the etching process. The morphology and optical properties of the porous layer were shown to have a significant effect on the short-circuit current and, accordingly, on the concentration of non-equilibrium charge carriers in the semiconductor wafer.

## 5. Conclusions

It has been shown in this paper that porous silicon of different thicknesses, pore diameters, and porosity can be effectively fabricated by the photo-assisted etching on a Si surface without external bias or metals. The morphology of porous layers can be modified by varying parameters such as etching duration, surface illumination intensity, and wafer resistivity. Charge carriers that occur in Si during etching under illumination and in the dark lead to black and brown silicon formation. The total current on the Si surface can reach 3 mA for *ρ* = 0.01 Ω·cm and 8000 lx illumination. The total current value is comparable to the current during electrochemical etching. Por-Si can be used to enhance optical absorption in the UV, visible, and near-IR spectra. The results obtained in this work will allow the development of technology to increase the efficiency of the solar cells at the p–n junction due to the formation of black silicon on the Si surface. Control of the porous layer thickness with a minimum reflection coefficient will be carried out by measuring the current using the short-circuit current.

## Figures and Tables

**Table 1:** Charge values for porous silicon samples.

Value	Illumination, lx	*ρ* = 0.01 Ω·cm	*ρ* = 1 Ω·cm	*ρ* = 12 Ω·cm
*Q_total_* (Equation (4))	0	1.535 ± 0.953	0.688 ± 0.282	0.437 ± 0.024
*Q’_Excess Carrier_* (*J(t))*		0.27	0.16	0.039
Δ*Q* (Equation (5))		1.265	0.528	0.398
*Q_total_*	460	2.02 ± 1.3	1.17 ± 0.043	0.86 ± 0.437
*Q’_Excess Carrier_*		0.348	0.37	0.496
Δ*Q*		1.674	0.8	0.364
*Q_total_*	8000	5.95 ± 4.475	2.95 ± 0.0062	1.77 ± 0.948
*Q’_Excess Carrier_*		5.28	2.05	1.64
Δ*Q*		0.67	0.9	0.13

**Table 2:** The roughness and the pore diameters of black and brown silicon.

№	Treatment duration, min	Illumination, lx	P, % (gravimetric analysis)	Roughness, nm (AFM)	d pores, nm (SEM)
1	10	460	14	22	10−100
2	60	460	60	50	25−125
3	60	8000	68	58	90–440
